# A Longitudinal Cohort Assessing the Carotid Intima-Media Thickness Progression and Cardiovascular Risk Factors in a Rural Black South African Community

**DOI:** 10.3390/jcm14031033

**Published:** 2025-02-06

**Authors:** Given R. Mashaba, Wendy N. Phoswa, Sogolo L. Lebelo, Solomon S. R. Choma, Eric Maimela, Kabelo Mokgalaboni

**Affiliations:** 1 Department of Life and Consumer Sciences, College of Agriculture and Environmental Sciences, University of South Africa, Science Campus, Private Bag X6, Florida, Roodepoort 1710, South Africa; given.mashaba@ul.ac.za (G.R.M.); phoswwn@unisa.ac.za (W.N.P.);; 2 DIMAMO Population Health Research Centre, University of Limpopo, Private Bag X1106, Sovenga 0727, South Africa; eric.maimela@ul.ac.za; 3 Department of Pathology, University of Limpopo, Private Bag X1106, Sovenga 0727, South Africa; solomon.choma@ul.ac.za

**Keywords:** carotid intima-media thickness, cardiovascular risk factors, diabetes mellitus, rural black South African community

## Abstract

**Background:** Diabetes mellitus [DM) is a fast-increasing non-communicable disease in South Africa, with a prevalence of 11.3%. The present study aimed to longitudinally investigate the association of carotid intima-media thickness [CIMT) progression and cardiovascular risk factors in the T2DM and non-DM rural black population of South Africa. **Methods:** This population-based retrospective cohort study was conducted in the Dikgale Mamabolo Mothiba Surveillance area between 2014 and 2023 by the Africa Wits INDEPTH Partnership for Genomic Research (AWI-Gen). The IBM Statistical Package for the Social Sciences version 27 was used to analyze data. The paired T-test was used to determine the mean differences between baseline and follow-up. Longitudinal estimates of the association of CIMT with CVD risk factors in the T2DM and non-DM groups were analyzed using linear mixed models. **Results:** The baseline mean age was 51.64 years. There was a significant increase in CIMT (left and mean CIMT), low-density lipoprotein-cholesterol (LDL-C), systolic blood pressure (SBP), diastolic blood pressure (DBP), and pulse rate in the T2DM and non-DM groups. In the T2DM group, there was a strong significant association between age (2.20 mm), LDL-C (4.30 mm), SBP (4.57 mm), and waist/hip ratio (0.24 mm) with CIMT progression. The non-DM group revealed a significant association between LDL-C (0.001 mm), SBP (1.41 mm), and CIMT progression. **Conclusion:** CIMT was associated with other main CVD-related risk factors (age, LDL-C, LDL-C/HDL-C ratio, TC/HDL-C ratio, waist/hip ratio, and SBP). CIMT progression was more pronounced in the T2DM group than non-DM, suggesting a higher risk of atherosclerosis and cardiovascular complications in T2DM individuals.

## 1. Introduction

According to the International Diabetes Federation (IDF), diabetes mellitus (DM) is a fast-increasing non-communicable disease globally, including in South Africa, where its prevalence is 11.3% [1]. In 2011, there were approximately 366 million people worldwide with DM, a number projected to rise to 552 million by 2030, making it the seventh leading cause of mortality globally [2]. A study by Hossain et al. [3] anticipates that the prevalence of DM will increase to 643 million (11.3%) by 2030 and 783 million (12.2%) by 2045. In recent years, the prevalence of type 2 diabetes mellitus (T2DM) has increased more rapidly in low- and middle-income countries (LMICs) compared to high-income countries (HICs), with about 79.4% of the global T2DM population living in LMICs [4]. The high prevalence of DM contributes to a growing economic burden [5]. These DM statistics call for more research to inform better management of the disease and prevention of associated complications.

The common complications associated with T2DM include cardiovascular diseases (CVDs), which are the cause of mortality among these individuals [6,7]. Over the years, researchers have been using carotid intima-media thickness (CIMT) as the main biomarker to predict the future development of CVDs [8]. While there are other biomarkers for atherosclerosis, CIMT directly measures early structural changes in the carotid artery, such as intima thickness and arterial stiffness [9]. In addition, the advancements in ultrasound technology have improved the reliability and validity of this CIMT test [10,11,12,13], making its results more usable in predicting CVDs. The traditional modifiable CVD risk factors, such as high blood pressure, dyslipidemia, and obesity, are reportedly associated with high CIMT [14,15,16]. Although CIMT measurement is considered non-invasive and convenient, it is not a readily available test in public health care facilities, especially in rural South Africa, making it difficult to monitor the extent of CIMT progression in people at risk of CVDs, including those with DM.

Theofilis et al. [17] reported that individuals with DM had higher mean CIMT and an increased burden of carotid plaque compared to healthy individuals. A higher CIMT in obesity may exacerbate the progression of CIMT as a predictor of endothelial dysfunction [18,19]. This is made worse by the interplay of the pro-inflammatory state, insulin resistance, hyperglycemia, dyslipidemia, and high blood pressure, which are associated with DM [20,21,22]. The Dikgale Mamabolobo Mothapo (DIMAMO) area, where the present study was conducted, has a high prevalence of T2DM [between 7% and 28% versus a national prevalence of 11.3%) [1,23,24], obesity [general obesity (35.4%) and central obesity (59.9%)], and hypertension (45.8% versus national prevalence of 38%)] [25,26,27,28]. The above-mentioned factors have been reported to have a negative impact on CIMT [29]. The DIMAMO area has a low socioeconomic status coupled with limited evidence of CIMT due to infrastructural challenges in primary health care, thus necessitating research that delves into CIMT progression. This would assist in monitoring cardiovascular risk, especially in individuals with T2DM. Furthermore, rural areas such as the DIMAMO area are less represented in cardiovascular research. Thus, this study aims to investigate the association between CIMT progression and CVD risk factors in DM individuals through a longitudinal study. Investigating the differences in CIMT progression and associated CVD risk factors between DM and healthy individuals will contribute to the existing body of knowledge and further provide insights for developing targeted interventions.

## 2. Materials and Methods

### 2.1. Study Design and Setting

This population-based retrospective cohort study was conducted amongst participants residing in the DIMAMO Surveillance area. This study was purposive in that participants who were part of the first phase of the cohort were targeted. Data were collected between 2014 (first cohort) and 2023 (second cohort) by the Africa Wits INDEPTH Partnership for Genomic Research (AWI-Gen). The first phase of the cohort included 1399 randomly selected participants aged 40 years and older. In the second phase of the cohort, about 673 participants were resurveyed using purposive sampling (Supplementary Figure S1). Of those surveyed, participants with missing variables of interest and outliers were removed. The total number of participants included in the present study was 410.

### 2.2. Ethics Approval and Participant’s Consent

The first phase of the cohort was approved by the University of Limpopo’s MEDUNSA Research Ethics Committee (MREC) (MREC/HS/195/2012:CR) in 2014. The University of Limpopo’s Turfloop Research Ethics Committee (TREC) approved the second phase of the cohort (TREC/264/2021:PG) in 2019. However, for the present study analysis, ethics approval was obtained from the University of South Africa’s College of Agriculture and Environmental Sciences Health Research Ethics Committee (REC-170616-051) and the DIMAMO Population Health Research Centre in 2023 and 2024, respectively. All patients provided written informed consent at the data collection level, indicating that the data would be used for future publication.

### 2.3. Participant’s Characteristics and Measurement

Demographic characteristics such as age, gender, and medical history were collected from the AWi-Gen questionnaire. Systolic blood pressure (SBP) and diastolic blood pressure (DBP) were recorded as the average of three measurements in the sitting position after 3 min rest, all measured in mmHg. Height (m) and weight (kg) were taken according to the standard protocols, with shoes removed and the participants wearing light clothing. Body mass index (BMI) was calculated as weight (kg) divided by the square of height (m).

Waist and hip measurements were taken according to the standard protocols, and the optimal cutoff values for waist circumference were 94 cm in males and 80 cm in females. Central obesity was defined as a WC of ≥80 cm for females and ≥94 cm for males. CIMT, subcutaneous adipose tissues (SATs), and visceral adipose tissues (VATs) were measured using LOGIC e-ultrasound as previously determined [30]. The optimal cutoff values for VAT were 6.5 and 5.0 cm for male and female, respectively. The subcutaneous adipose tissue cutoff value was 1.82 cm in males and 1.46 cm in females. Individuals with a VAT of above 6.5 and 5.0 cm for males and females were considered centrally obese. Individuals with an SAT above 1.82 and 1.46 cm for males and females were considered centrally obese.

A qualified research nurse collected peripheral blood samples. Biochemical parameters, including total cholesterol (TC), triglycerides (TGs), low-density lipoprotein (LDL-C), high-density lipoprotein (HDL-C), and glucose, were determined with a commercially available assay kit on the Beckman Coulter platform (Beckman Coulter Inc., Brea, CA, USA) clinical chemistry analyzer. T2DM was defined as fasting blood glucose (FBG) ≥7.0 mmol/L in the cohort exam or self-reported history of T2DM diagnosed by a physician. CIMT progression was defined as a change in CIMT during follow-up compared with the baseline. This was performed by subtracting follow-up CIMT values from the baseline values.

### 2.4. Data Analysis

Participants with missing data were removed. Baseline outliers were removed using the interquartile range (IQR) method through Microsoft Excel 2016. Briefly, the first (Q1) and third (Q3) quartiles were determined and used to determine the interquartile range (IQR = Q3 − Q1). We further used the formulae Q3 + (1.5 × IQR) and Q1 − (1.5 × IQR) to determine the upper and lower bounds, respectively. Variable values that fell outside the lower and upper bounds were removed from the data set. The removal of outliers and exclusion of participants with incomplete data were used to ensure the sensitivity of the analysis and the robustness of the results [31]. A categorical value of change in CIMT was created to identify cases of increased and decreased CIMT. This categorical variable was used along with baseline values of CVD risk factors in the sensitivity analysis. The data were analyzed using the IBM Statistical Package for the Social Sciences (SPSS) version 27. The paired T-test was used to determine mean differences between baseline and follow-up. Longitudinal estimates of the association of mean CIMT with CVD risk factors in the DM and non-DM groups were analyzed using linear mixed models. A *p*-value of less than 0.05 was considered statistically significant.

## 3. Results

Table 1 presents the baseline characteristics of participants. The mean age at baseline was 51.64 years, without a significant difference between males and females. There was no significant difference in CIMT levels between males and females, with *p*-values of 0.285, 0.992, and 0.549 for the left, right, and mean CIMT, respectively. Compared to males, the majority of females had significantly an elevated TC (*p* < 0.001) and TC/HDL-C ratio (*p* = 0.002). However, there was no significant difference between males and females in terms of HDL-C, LDL-C, and Trig/HDL-C. However, TG and LDL-C/HDL-C ratios were more elevated in males than females, with *p*-values of 0.027 and 0.003, respectively. Markers of central obesity, such as visceral fat, subcutaneous fat, and waist circumference, were significantly elevated in females than in males, with *p*-values of 0.039, <0.001, and 0.003, respectively. However, males had a significantly higher waist/hip ratio than females (*p* = 0.020). The mean BMI at baseline was 28.37 ± 7.57, with females having a significantly higher BMI (30.83 ± 7.30) than males (22.27 ± 3.90), *p* = 0.005. The prevalence of T2DM was 45.4%, with no significant difference between males and females.

There was a significant increase in CIMT (left and mean CIMT) in both groups [T2DM group (0.043 ± 0.15, *p* < 0.001 and 0.02 ± 0.12, *p* = 0.024) and non-DM group (0.02 ± 0.13, *p* = 0.006 and 0.021 ± 0.11, *p* = 0.046)], indicating a progression in arterial wall thickness between baseline and follow up. However, the T2DM group showed a slightly larger increase in left CIMT than the non-DM group. We noted a significant increase in lipid profile and lipids in both groups, with the T2DM group having slightly higher increases than the non-DM group. The T2DM group had significantly high values in terms of change in visceral fat decrease (1.01 ± 2.26), compared to the non-DM group, which had a (0.56 ± 2.47) significant reduction in visceral fat. There was no significant change in subcutaneous fat, waist circumference, and BMI in the T2DM group. The same was observed in the non-DM group except for the BMI, in which we noted a (0.576 ± 3.30) significant increase between baseline and follow-up (*p* = 0.007). Blood pressure measurements were also increased in both groups, with the T2DM group showing slightly increased SBP and DBP compared to the non-DM group (Table 2).

Table 3 compares baseline versus follow-up by gender in the T2DM group. The left CIMT significantly increased in both the male and female participants, with *p*-values of 0.010 and 0.023, respectively. However, neither gender showed significant change in the right CIMT. We only noted a statistically significant increase in the mean CIMT in the male participants (*p* = 0.024). There was an increase in HDL-C and a decrease in LDL/HDL ratio in both genders; however, this was not significant. Both genders showed a significant increase in TC with a *p* < 0.001. Additionally, there was a significant increase in triglycerides (*p* = 0.005) and TC/HDL-C (*p* < 0.001) in the female but not in the male participants. We also noted a significant decrease in visceral fat in both males (0.56 ± 2.47, *p* < 0.001) and females (1.01 ± 2.26, *p* < 0.001) participants. The waist/hip ratio significantly decreased in females (*p* = 0.042) but not in males. The SBP was a significant increase in both participants (females: *p* < 0.001, males: *p* = 0.044); however, SBP significantly decreased only in the female participants (*p* < 0.001).

In the non-DM group, as shown in Table S1, the left and mean CIMT were significantly increased in males but not in females, with *p*-values of 0.002 and 0.009, respectively. We noted a decrease in HDL-C and LDL/HDL-C in both groups. However, this was not significant. Total cholesterol was significantly increased in both groups (*p* < 0.001, respectively), while LDL-C was significantly increased in male participants only (*p* < 0.001). The remaining lipids and lipid ratios (Trig, TC/LDL-C, and Trig/LDL-C ratio) significantly increased in the female participants but not in male participants. We noted a significant decrease in visceral fat in both groups (*p* = 0.029 for females, *p* < 0.001 for males) and a significant reduction in subcutaneous fat in male participants. The BMI significantly increased in females (*p* = 0.009) but not in males. There was a significant increase in SBP (*p* < 0.001) and a significant decrease in DBP (*p* = 0.035) in female participants but not in male participants.

**Figure 1:**
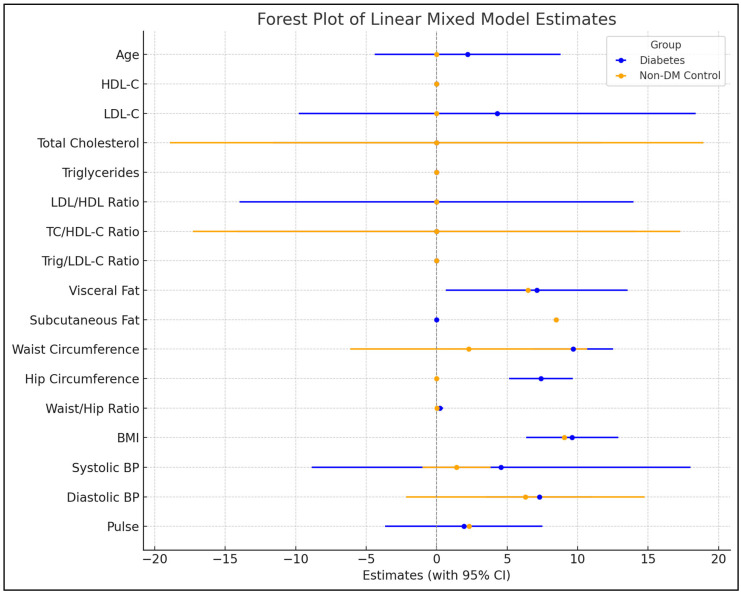
Longitudinal estimates of the association of CIMT with CVD risk factors in the T2DM and non-DM groups using linear mixed models.

In the T2DM group, age was significantly associated with CIMT, with an effect estimate of 2.20 mm (*p* < 0.001); this suggests that CIMT increases as the T2DM group ages. However, there was no significant association between change in CIMT and age in the non-DM group (*p* = 0.372). We observed no significant association between HDL-C, TC, TG, and the trig/HDL-C ratio with CIMT in both groups. In the T2DM group, LDL-C was significantly associated with CIMT progression, with an estimate of 4.30 mm (*p* < 0.001), suggesting a strong association between LDL-C and CIMT progression. However, there was a weak association between LDL-C and CIMT progression in the non-DM group due to the estimate of 0.001 mm (*p* < 0.001). Both groups had a significant association between LDL/HDL and TC/HDL ratios with CIMT; however, the effect size was small, 0.001 mm, *p* < 0.001. Regarding measures of obesity, we observed no significant association between the BMI, visceral and subcutaneous fat, waist, and hip circumference with CIMT progression in both groups (*p* ˃ 0.05). In contrast, we noted a significant association between the waist/hip ratio and CIMT progression, with an effect estimate of 0.237 mm (*p* = 0.001) in the T2DM group. However, no association was noted in the non-DM group (*p* = 0.591). Regarding blood pressure measurements, we found no association between DBP and CIMT progression. Interestingly, we demonstrate a significant association between SBP and CIMT progression in both groups. However, this was more pronounced in T2DM [4.572 mm, *p* = 0.019) compared to the non-DM group (1.411 mm, *p* = 0.004) (Figure 1 and Table S2).

## 4. Discussion

Carotid intima-media thickness progression in individuals with diabetes is a complex phenomenon influenced by various factors such as age, gender, lipid levels, BMI, visceral fat, and blood pressure. This study investigated the differences in CIMT progression and associated CVD risk factors between the T2DM and non-DM groups. This study found that factors such as lipid profile, age, hypertension, and obesity promoted the rapid progression of CIMT in T2DM compared to the non-DM group. This aligns with a review on the subject, which found that CIMT was significantly higher in patients with T2DM than those without T2DM [32]. Age is a significant factor influencing CIMT progression in individuals with diabetes. Our study found that the mean age at baseline was 51.64 years, with no significant difference between males and females. A study by Lorenz et al. [33] reported that the association between CIMT and cardiovascular risk was stronger in people aged 50. The initial manifestation of elevated CIMT, the progression of which leads to plaque formation and vascular narrowing, is reportedly part of the aging process and can present itself even in non-DM patients [34]. As people age, their blood vessels tend to be more rigid and less elastic, resulting in endothelial dysfunction [35,36]. In addition, oxidative stress, systemic inflammation, and impaired endothelial function increase with age [37,38]. Altogether, these factors are more pronounced in diabetes. The present study found a positive significant association between age and CIMT progression in the T2DM group; however, there was no association in the non-DM group. This suggests that an increase in age predisposes T2DM individuals of the black South African demographic to CIMT thickening and atherosclerosis. This necessitates frequent CIMT monitoring and early detection of CIMT in this demographic to prevent the development of atherosclerosis. Although there is limited evidence investigating the progression of CIMT in the study area, another study with the same methodological approach found that CIMT changed by approximately 0.09 to 0.04 mm annually in people with DM [39].

This study showed no significant difference in CIMT levels between males and females at baseline (Table 2). The same trend was observed in right CIMT in both genders at the follow-up stage. In contrast, there was a significant increase in left and mean CIMT at follow-up in males and increased left CIMT in females in the T2DM group. Consistently, in the non-DM group, we observed a significant increase in left and mean CIMT in males without significant change in female participants. It is worth noting that while the progression of CIMT was noted in both groups in males, this was more pronounced in the T2DM than in the non-DM group. These findings suggest that males, compared to females, have an increased risk of CIMT progression and the development of atherosclerosis. It is also important to indicate that the present study predominantly constituted females. Therefore, it is possible that this could have influenced the results due to gender imbalance, as males and females have different cardiovascular risk profiles [40]. As a result, the lower number of males in this study may have exaggerated the differences in CIMT progression between genders. Gender-related risks for diabetes vary from one population to another, with males being reported to have a higher risk for DM-related vascular complications such as atherosclerosis than females [41]. In males, the risk of atherosclerosis seems to increase after the age of 45 years, while in females, the risk rises after the age of 55 years [42]. The development of atherosclerosis in males as they age could be attributed to a decrease in testosterone levels with age. For instance, low serum testosterone concentration has been reported as an independent risk factor in the development of atherosclerosis and other CVD [43]. Briefly, as males age, testosterone decreases, which promotes the accumulation of small dense LDL (sdLDL), resulting in vascular inflammation and further promoting plaque formation [44]. Additionally, due to the susceptibility of sdLDL to oxidation, they attract macrophages, resulting in the formation of foam cells and the buildup of plaque in arteries [45]. Moreover, the overall risk of cardiovascular events has been reported to be significantly higher in patients with high levels of LDL-C [46]. Notably, the male participants in the study area have a higher prevalence of cardiovascular risk due to lifestyle choices such as increased alcohol consumption and tobacco use [27].

The lipid profiles and their ratios were also associated with CIMT progression in this study. For instance, for each unit increase in LDL, CIMT progressed by 4.30 mm in T2DM and 0.001 mm in the non-DM group. This suggests that LDL-C has a stronger association with CIMT progression in T2DM than in the non-DM group. Additionally, the T2DM group showed a significant increase in TC/HDL-C among female participants. In the non-DM group, lipid ratios such as TC/LDL-C and Trig/LDL-C were significantly elevated in the female participants but not in the male participants. However, in both T2DM and non-DM groups, we observed no significant association between HDL-C, TC, Trig, and the Trig/HDL-C ratio with CIMT. Similarly, no significant association was observed between the Trig/HDL-C ratio and CIMT in either group. However, both groups had a significant yet weak association between the LDL/HDL ratio and CIMT progression. In addition to individual serum lipid levels, lipid ratios have been demonstrated to be more favoured as predictors for cardiovascular risk [47,48]. This is because the serum lipid ratios are a measure of the relative concentration of atherogenic lipids [TC, Trig, and LDL-C] to anti-atherogenic lipids [HDL-C] [47,48]. Lipoprotein (a) (Lp (a) is deemed as an emerging indicator of cardiovascular risk due to its involvement in fibrinolysis, vascular smooth muscle cell proliferation, monocyte adhesion, and activation of pro-inflammatory status [49]. Although not included in this study, it may be important to consider Lp (a) in cardiovascular risk assessment and CIMT progression, particularly in underrepresented populations.

CIMT progression was also found to be negatively influenced by obesity. Obesity may worsen the progression of CIMT as a predictor of endothelial dysfunction [50]. Moreover, obesity increases the risk of developing T2DM [18,19]. This occurs through the interplay of the pro-inflammatory cytokines, insulin resistance, and hyperglycemia [20,21,22]. The female baseline BMI was significantly higher than that of male participants. Generally, females have a higher fat percentage than males due to hormonal differences, which promote fat accumulation in the buttock area [51]. Although there was no significant association between the BMI and CIMT progression, the observable difference showed that for each unit increase in the BMI, CIMT increased by 9.612 mm in the T2DM and 9.064 mm in the non-DM group. When the analysis was conducted based on diabetes status, we noted that the BMI was significantly higher in the non-DM group without any significant change in T2DM. In most cases, especially in South African primary health care, the BMI is considered when diagnosing obesity. However, the BMI is a simple ratio of weight to height and does not distinguish between muscle and fat mass [52,53]. The BMI can sometimes fail to reflect the body fat composition accurately [53]. Therefore, to understand fat distribution, other reliable tests, such as ultrasound measurements of visceral fat and waist circumference, are employed [54]. In this study, visceral fat was lower in both genders but more pronounced in female than male participants. Additionally, based on diabetes status, the T2DM group had lower visceral fat than the non-DM group. High waist circumference indicates an excess accumulation of visceral fat, which is more metabolically active and harmful than subcutaneous fat [55]. In this study, no differences were noted regarding waist circumference in both genders and groups. Moreover, no association was found between waist circumference and CIMT progression. These suggest that BMI alone cannot be reliable as a marker of obesity in T2DM. General and central obesity predisposes individuals to insulin resistance, inflammation markers, and dyslipidemia [56]. This subsequently results in cardiovascular-related complications. Although general and central obesity predisposes individuals to similar risks, these populations are reported to be more metabolically active and release more inflammatory and free fatty acid markers into the bloodstream [57]. Metabolically healthy obese individuals have a lower risk of CVD and mortality compared with those with metabolically unhealthy obesity [58]. Interestingly, while metabolic activity induces systemic inflammation, higher metabolic activity has been reported to improve lipid profiles and insulin sensitivity [59,60].

In the present study, we found that for each unit increase in visceral fat, CIMT increased by 7.102 mm in the T2DM group and 6.475 mm in the non-DM group. Although not significant, the results of the present study align with a cross-sectional study conducted in the same population that found that T2DM patients who are centrally obese are at high risk of developing CVD complications [27]. In addition, there was a significant decrease in visceral fat in the present study, with the T2DM group having a pronounced change in visceral fat compared to the non-DM group. A study by Kim et al. [61] conducted on Korean individuals with DM reported that visceral fat declined by 5.2 ± 13.5 mm in males and 3.4 ± 10.5 mm in females during a two-year observation, with the progression of CIMT only significant for females. On the other hand, Eickemberg et al. [62], in a cohort conducted in Brazil, reported that abdominal adiposity had a positive association with CIMT progression in both genders (males: OR = 1.47, 95%CI: 1.22–1.77, females: OR = 1.38; 95%CI: 1.17–1.64).

The results of the present study are supported by previous studies that indicate that central obesity in diabetes negatively impacts CIMT progression; however, the impact varies in different demographics [61,62,63]. The mechanism behind this association is attributed to hyperglycemia. The existing evidence shows that insulin deficiency impairs cells’ ability to use glucose as an energy source [64]; consequently, high glucose concentrations in the bloodstream lead to hyperglycemia [64]. In the absence of sufficient insulin, the body fails to effectively use glucose for energy, so it shifts to burning fat for fuel [65]. This can result in reduced abdominal fat storage [65]. Similarly, a cell’s inability to use glucose as an energy source that results from the lack of insulin can trigger reliance on fat stores as the only energy supply [65,66]. The continuous dependence on fat may induce the release of ketones into the bloodstream, which leads to the chronic condition of ketoacidosis [65,66].

Blood pressure measures were also increased in both groups, with the T2DM group showing slightly increased SBP and DBP compared to the non-DM group. The SBP was significantly associated with CIMT progression in the T2DM group and showed a 1.411 mm increase in CIMT in the non-DM group. Although DBP was associated with CIMT progression by 7.281 mm in the T2DM and 6.308 mm in the non-DM group, this was not significant. It is worth noting that elevated blood pressure is a central feature in diabetes independent of age, adiposity, and renal function [67]. Blood pressure and diabetes contribute to CIMT progression, and this is exacerbated by the progression of hypertension in diabetes [68]. Insulin affects hypertension by acting as a peripheral vasodilator through endothelial nitric oxide-dependent pathways and increasing sodium retention in the renal system [69]. Consistently, hypertension impairs the endothelium, reducing nitric oxide production and further contributing to arterial stiffness, CIMT thickening, and atherosclerosis [70,71]. On the other hand, hypertension induces inflammation by promoting the production of pro-inflammatory cytokines. These cytokines stimulate the proliferation of vascular smooth muscle cells, resulting in arterial wall thickening and CIMT progression [72,73,74,75]. Therefore, it is important to control hypertension in diabetes to curb the rising risk of atherosclerosis and associated complications.

### Strengths and Limitations

The present study was conducted on individuals of the black race residing in the DIMAMO area, thus limiting the generalizability of the findings to other populations. However, this rural population remains underrepresented in cardiovascular research. Therefore, the present study presents insight into the risk faced by this group. The present study was a retrospective cohort; only baseline CIMT and CIMT were measured after 5 years, thus making it difficult to track CIMT every year. However, the changes in this 5-year period were determined using data from the baseline (first cohort) and the follow-up period (end of the second cohort). Additionally, the location where this study was conducted is of low socioeconomic status, with infrastructural limitations in primary health care, such as the routine measurement of CIMT, which may limit the applicability of the findings to this population. The gender imbalance in this study could have contributed to some differences as there were more female than male participants. Although inflammation and genetic predispositions play a role in CIMT progression and atherosclerosis, they were not assessed in this study.

## 5. Conclusions

This study found that cardiovascular risk factors such as age, LDL-C, LDL-C/HDL-C ratio, TC/HDL-C ratio, waist/hip ratio, and SBP are significantly associated with CIMT progression in T2DM individuals. Although other risk factors (LDL-C, SBP, and waist-hip ratio) were significantly increased in both groups, their impact on CIMT progression was more pronounced in the T2DM group, indicating a higher cardiovascular risk for T2DM individuals. Hence, these factors should be taken into consideration in the management of T2DM. We therefore recommend more frequent CIMT measurements in future studies to better evaluate temporal changes. We also suggest future studies with gender-balanced sample sizes focus on inflammatory biomarkers due to their contribution to the development of atherosclerosis.

## Figures and Tables

**Table 1:** Baseline characteristics of included participants.

Variables	Total (n = 410)	Male (n = 118)	Female (n = 292)	*p*-Value
Age (years)	51.64 ± 6.67	51.43 ± 7.0	51.73 ± 6.54	0.562
Diabetes, n (%)	186 (45.4)	60 (50.8)	186 (45.5)	0.189
**CIMT measurement**				
Left CIMT (mm)	0.63 ± 0.098	0.64 ± 0.11	0.63 ± 0.09	0.285
Right CIMT (mm)	0.65 ± 0.10	0.65 ± 0.10	0.65 ± 0.10	0.992
Mean CIMT (mm)	0.64 ± 0.09	0.64 ± 0.09	0.64 ± 0.09	0.549
**Lipid profile**				
HDL-C (mmol/L)	1.19 ± 0.32	1.19 ± 0.37	1.19 ± 0.31	0.058
LDL-C (mmol/L)	2.59 ± 0.84	2.62 ± 0.85	2.57 ± 0.83	0.336
TC (mmol/L)	4.17 ± 1.19	4.09 ± 1.07	4.20 ± 1.24	<0.001 **
Trig (mmol/L)	1.11 ± 0.60	1.17 ± 0.67	1.09 ± 0.57	0.027 *
LDL/HDL ratio	2.40 ± 1.24	2.53 ± 1.48	2.34 ± 1.19	0.003 *
TC/LDL-C ratio	3.68 ± 1.18	3.67 ± 1.17	3.68 ± 1.19	0.002 *
Trig/LDL-C ratio	1.03 ± 0.70	1.13 ± 0.90	0.98 ± 0.59	0.054
**Obesity measurements**				
Visceral fat (cm)	6.49 ± 2.04	6.28 ± 1.79	6.57 ± 2.13	0.039 *
Subcutaneous fat (cm)	1.88 ± 1.04	0.97 ± 0.49	2.24 ± 0.98	<0.001 **
Waist circumference (cm)	90.64 ± 14.75	81.97 ± 11.55	94.15 ± 14.46	0.003 *
Hip circumference (cm)	104.11 ± 15.06	91.34 ± 8.95	109.28 ± 13.90	<0.001
Waist/hip ratio	0.87 ± 0.08	0.90 ± 0.06	0.86 ± 0.08	0.020 *
BMI (kg/m^2^)	28.37 ± 7.57	22.27 ± 3.90	30.83 ± 7.30	0.005 *
**Blood pressure measurement**				
SBP (mmHg)	125.50 ± 17.77	123.96 ± 17.08	126.12 ± 18.04	0.068
DBP (mmHg)	80.95 ± 11.32	78.03 ± 10.47	82.13 ± 11.46	0.016 *
Pulse (mmHg)	65.75 ± 10.81	62.84 ± 11.34	66.93 ± 10.37	<0.001 **

Data were presented in terms of mean ± standard deviation or number (%). *p*-value: significance of the results (* *p* < 0.05, ** *p* < 0.001). CIMT: carotid intima-media thickness, BMI: body mass index, SBP: systolic blood pressure, DBP: diastolic blood pressure, HDL-C: high-density lipoproteins cholesterol. LDL-C: low-density lipoproteins cholesterol. TC: total cholesterol. Trig: Triglycerides.

**Table 2:** Within-group comparison of baseline versus follow-up in the T2DM group and non-DM group.

	T2DM Group				Non-DM Group			
Variables	Baseline	Follow-Up	Difference	*p*-Value	Baseline	Follow-Up	Difference	*p*-Value
Age (years)	51.98 ± 7.00	58.00 ± 6.94	6.02 ± 2.21	<0.001 **	52.28 ± 7.27	58.06 ± 7.42	5.78 ± 1.60	<0.001 *
**CIMT measurement**								
Left CIMT (mm)	0.65 ± 0.13	0.69 ± 0.15	0.04 ± 0.15	<0.001 **	0.66 ± 0.13	0.68 ± 0.13	0.02 ± 0.13	0.006 *
Right CIMT (mm)	0.67 ± 0.13	0.67 ± 0.14	0.00 ± 0.15	0.747	0.67 ± 0.13	0.67 ± 0.13	0.00 ± 0.13	0.545
Mean CIMT (mm)	0.66 ± 0.12	0.68 ± 0.12	0.02 ± 0.12	0.024 *	0.66 ± 0.12	0.68 ± 0.12	0.02 ± 0.12	0.046 *
**Lipid profile**								
HDL-C (mmol/L)	1.21 ± 0.41	1.26 ± 0.38	0.05 ± 0.37	0.043 *	1.17 ± 0.39	1.22 ± 0.36	0.05 ± 0.33	0.268
LDL-C (mmol/L)	2.70 ± 1.06	2.88 ± 0.98	0.18 ± 0.95	0.008 *	2.75 ± 0.94	2.86 ± 0.98	0.11 ± 0.92	0.051
TC (mmol/L)	4.17 ± 1.30	4.81 ± 1.26	0.64 ± 0.09	<0.001 *	4.11 ± 1.12	4.76 ± 1.08	0.65 ± 1.05	<0.001 **
Trig (mmol/L)	1.13 ± 0.62	1.27 ± 0.72	0.14 ± 0.05	<0.001 *	1.07 ± 0.53	1.27 ± 0.65	0.20 ± 0.68	<0.001 **
LDL/HDL ratio	2.78 ± 2.56	2.49 ± 1.12	−0.29 ± 0.16	0.074	2.75 ± 2.68	2.56 ± 1.19	−0.19 ± 2.67	0.248
TC/LDL-C ratio	3.72 ± 1.41	4.05 ± 1.29	0.33 ± 1.53	0.002 *	3.75 ± 1.25	4.19 ± 1.45	0.44 ± 1.44	<0.001 **
Trig/LDL-C ratio	1.06 ± 0.74	1.10 ± 0.68	0.04 ± 0.05	0.417	1.02 ± 0.69	1.12 ± 0.64	0.10 ± 0.75	0.023 *
**Obesity measurements**								
Visceral fat (cm)	6.58 ± 2.10	5.57 ± 2.26	−1.01 ± 2.26	<0.001 **	6.61 ± 2.69	6.06 ± 2.21	−0.55 ± 2.47	<0.001 **
Subcutaneous fat (cm)	1.83 ± 1.06	1.88 ± 1.27	0.05 ± 1.16	0.553	1.94 ± 1.06	1.97 ± 1.19	0.03 ± 1.06	0.617
Waist circumference (cm)	90.84 ± 15.45	91.36 ± 14.86	0.52 ± 8.96	0.388	91.61 ± 15.56	92.42 ± 14.30	0.81 ± 8.89	0.149
Hip circumference (cm)	103.48 ± 16.27	105.72 ± 17.64	2.24 ± 10.29	0.002 **	104.93 ± 15.68	106.36 ± 16.27	1.43 ± 8.61	0.008 *
Waist/hip ratio	0.88 ± 0.08	0.87 ± 0.09	−0.01 ± 0.08	0.040 *	0.87 ± 0.08	0.87 ± 0.07	0.00 ± 0.08	0.604
BMI (kg/m^2^)	28.29 ± 8.20	28.64 ± 8.45	0.35 ± 3.33	0.119	28.92 ± 8.15	29.49 ± 8.03	0.57 ± 3.30	0.007 *
**Blood pressure measurement**								
SBP (mmHg)	127.14 ± 18.87	132.80 ± 20.87	5.66 ± 21.77	<0.001 *	126.26 ± 19.16	130.41 ± 21.55	4.15 ± 20.11	0.001 *
DBP (mmHg)	79.23 ± 10.86	81.49 ± 12.27	2.26 ± 11.29	0.003 *	81.17 ± 12.46	79.63 ± 11.21	−1.54 ± 12.35	0.046 *
Pulse (mmHg)	65.28 ± 10.91	69.51 ± 10.54	4.23 ± 10.84	<0.001 *	67.33 ± 12.40	70.45 ± 11.55	3.12 ± 14.13	<0.001 *

Data were presented in terms of mean ± standard deviation. *p*-value: significance of the results (* *p* < 0.05, ** *p* < 0.001). CIMT: carotid intima-media thickness, BMI: body mass index, SBP: systolic blood pressure, DBP: diastolic blood pressure, HDL-C: high-density lipoproteins cholesterol. LDL-C: low-density lipoproteins cholesterol. TC: total cholesterol. Trig: Triglycerides. T2DM: type 2 diabetes mellitus.

**Table 3:** Within-group comparison of baseline versus follow-up by gender in the T2DM group.

	Females				Males			
Variables	Baseline	Follow-Up	Difference	*p*-Value	Baseline	Follow-Up	Difference	*p*-Value
Age (years)	52.35 ± 7.06	58.39 ± 7.00	6.05 ± 2.39	<0.001 **	51.21 ± 6.90	57.20 ± 6.78	5.99 ± 1.79	<0.001 **
**CIMT measurement**								
Left CIMT (mm)	0.65 ± 0.12	0.68 ± 0.15	0.03 ± 0.14	0.023 *	0.66 ± 0.15	0.66 ± 0.14	0.05 ± 0.15	0.010 *
RightCIMT(mm)	0.18 ± 0.14	0.67 ± 0.14	0.00 ± 0.15	0.691	0.66 ± 0.14	0.68 ± 0.13	0.02 ± 0.14	0.220
MeanCIMT(mm)	0.66 ± 0.13	0.67 ± 0.12	0.01 ± 0.12	0.260	0.66 ± 0.14	0.69 ± 0.02	0.03 ± 0.13	0.024 *
**Lipid profile**								
HDL-C(mmol/L)	1.21 ± 0.39	1.25 ± 0.36	0.04 ± 0.35	0.158	1.21 ± 0.46	1.28 ± 0.42	0.07 ± 0.42	0.148
LDL-C (mmol/L)	2.73 ± 1.04	2.84 ± 0.96	0.11 ± 0.96	0.171	2.66 ± 1.09	2.96 ± 1.02	0.30 ± 0.92	0.008 *
TC (mmol/L)	4.27 ± 1.37	4.96 ± 1.24	0.69 ± 1.32	<0.001 **	3.97 ± 1.14	4.50 ± 1.25	0.53 ± 1.18	<0.001 *
Trig (mmol/L)	1.11 ± 0.67	1.29 ± 0.70	0.18 ± 0.76	0.005 *	1.15 ± 0.62	1.23 ± 1.24	0.09 ± 0.74	0.327
LDL/HDL ratio	2.73 ± 2.58	2.46 ± 1.08	−0.27 ± 2.31	0.156	2.86 ± 2.55	2.56 ± 0.14	−0.30 ± 2.33	0.279
TC/LDL-C ratio	3.73 ± 1.28	4.17 ± 1.21	0.46 ± 1.38	<0.001 **	3.70 ± 1.67	3.78 ± 1.42	0.09 ± 1.81	0.679
Trig/LDL-C ratio	1.02 ± 0.70	1.12 ± 0.69	0.11 ± 0.76	0.095	1.13 ± 0.83	1.06 ± 0.65	0.07 ± 0.84	0.404
**Obesity measurements**								
Visceral fat (cm)	6.69 ± 2.18	5.90 ± 2.37	−0.79 ± 2.31	<0.001 **	6.36 ± 1.19	4.89 ± 1.85	−1.47 ± 2.06	<0.001 **
Subcutaneous fat (cm)	2.22 ± 1.04	2.21 ± 1.40	−0.01 ± 1.38	0.444	1.01 ± 0.46	1.18 ± 0.42	0.18 ± 0.37	<0.001 **
Waist circumference (cm)	94.75 ± 15.51	95.15 ± 14.76	0.38 ± 9.45	0.310	82.72 ± 11.80	83.53 ± 11.72	0.81 ± 7.89	0.391
Hip circumference (cm)	109.40 ± 15.75	111.77 ± 17.45	2.37 ± 11.55	0.007 *	91.23 ± 8.79	93.18 ± 9.66	1.94 ± 7.07	0.023 *
Waist/hip ratio	0.87 ± 0.08	0.86 ± 0.08	−0.01 ± 0.09	0.042 *	0.90 ± 0.07	0.90 ± 0.08	0.00 ± 0.06	0.256
BMI (kg/m^2^)	31.20 ± 8.15	31.62 ± 8.35	0.49 ± 3.73	0.087	22.26 ± 3.87	22.47 ± 4.31	0.21 ± 2.31	0.437
**Blood pressure measurement**								
SBP (mmHg)	128.64 ± 18.93	134.44 ± 20.91	5.80 ± 21.71	<0.001 **	124.05 ± 18.51	129.42 ± 20.54	5.37 ± 22.04	0.044 *
DBP (mmHg)	83.56 ± 12.42	79.70 ± 11.07	−3.84 ± 11.13	<0.001 **	77.22 ± 10.86	78.25 ± 10.44	1.04 ± 10.98	0.430
Pulse (mmHg)	66.60 ± 10.48	70.51 ± 10.45	3.91 ± 10.53	<0.001 **	62.55 ± 11.37	67.46 ± 10.50	4.91 ± 11.51	<0.001 **

Data were presented in terms of mean ± standard deviation. *p*-value: significance of the results (* *p* < 0.05, ** *p* < 0.001). CIMT: carotid intima-media thickness, BMI: body mass index, DBP: diastolic blood pressure, SBP: systolic blood pressure, HDL-C: high-density lipoproteins cholesterol, LDL-C: low-density lipoproteins cholesterol, TC: total cholesterol, Trig: Triglycerides, T2DM: type 2 diabetes mellitus.

## Data Availability

The data presented in this study are available on request from the corresponding author. The data are not publicly available due to privacy or ethical restrictions.

## Supplementary Materials

The following supporting information can be downloaded at https://www.mdpi.com/article/10.3390/jcm14031033/s1, Figure S1: Flow diagram showing participant’s recruitment in phases 1 and 2, Table S1: Within-group comparison of baseline versus follow-up by gender in the non-DM group, Table S2: Longitudinal estimates of the association of CIMT with CVD risk factors in T2DM group and non-DM controls using linear mixed models.
