# Supplementary material for: A Longitudinal Cohort Assessing the Carotid Intima-Media Thickness Progression and Cardiovascular Risk Factors in a Rural Black South African Community
Source: J Clin Med. 2025 Feb 6;14(3):1033. doi: 10.3390/jcm14031033 (PMC11818556; doi:10.3390/jcm14031033)
Supplement: Supplementary file 1 [file jcm-14-01033-s001.zip › jcm-3350336-supplementary.pdf]

A Longitudinal Cohort Assessing the Carotid Intima-Media Thickness Progression and Cardiovascular Risk Factors in a Rural Black South African Community

Given R. Mashaba [1,2], Wendy N. Phoswa [1], Sogolo L. Lebelo [1], Solomon S. R. Choma [3], Eric Maimela [2] and Kabelo Mokgalaboni [1,*]

[1]Department of Life and Consumer Sciences, College of Agriculture and Environmental Sciences, University of South Africa, Science Campus, Private Bag X6, Florida, Roodepoort 1710, South Africa; given.mashaba@ul.ac.za (G.R.M.); phoswwn@unisa.ac.za (W.N.P.)

[2]DIMAMO Population Health Research Centre, University of Limpopo, Private Bag X1106, Sovenga 0727, South Africa; eric.maimela@ul.ac.za

[3]Department of Pathology, University of Limpopo, Private Bag X1106, Sovenga 0727, South Africa; solomon.choma@ul.ac.za

Correspondence:

Email: mokgak@unisa.ac.za

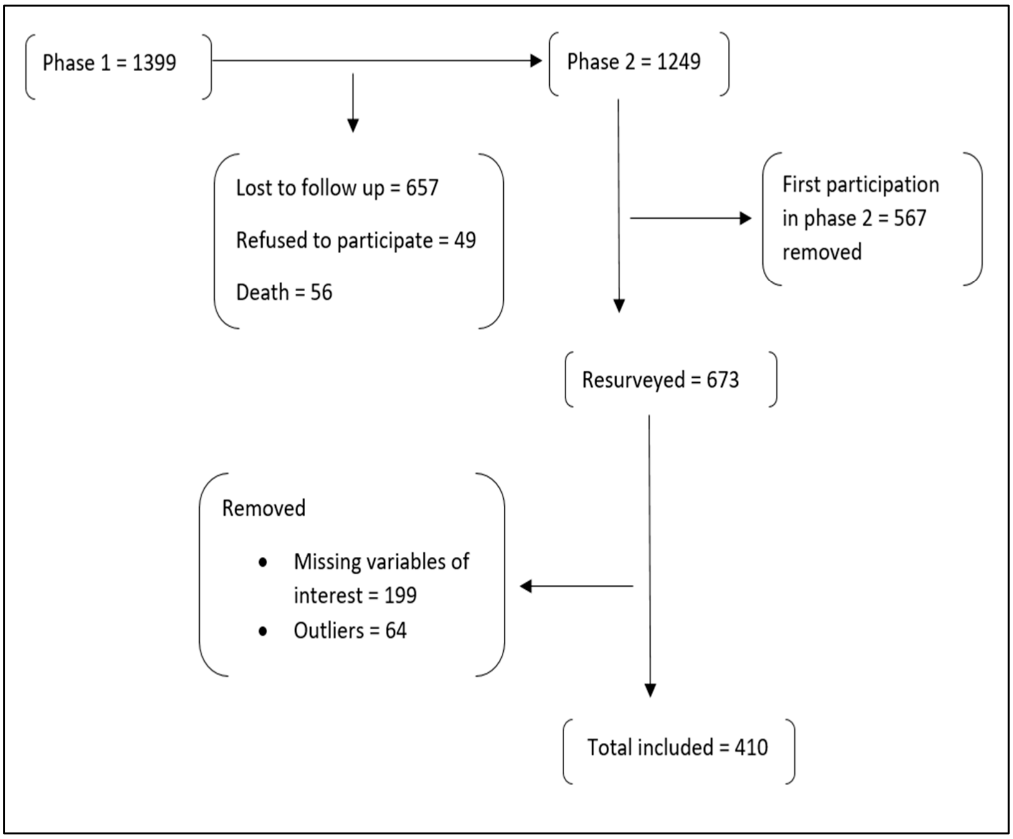

Figure S1. Flow diagram showing participant's recruitment in phases 1 and 2.

Table S1: Within-group comparison of baseline versus follow-up by gender in the non-DM group

	Females				Males			
**Variables**	**Baseline**	**Follow-up**	**Difference**	**P-value**	**Baseline**	**Follow-up**	**Difference**	**P-value**
Age	52.07±6.10	57.96±7.69	5.88±1.76	<0.001*	52.88±8.02	58.36±7.82	5.48±0.99	<0.001**
**CIMT measurement**								
Left CIMT	0.65±0.12	0.66±0.12	0.01±0.13	0.153	0.68±0.15	0.74±0.14	0.06±0.13	0.002*
Right CIMT	0.65±0.13	0.66±0.11	0.01±0.12	0.912	0.69±0.15	0.72±0.14	0.03±0.02	0.124
Mean CIMT	0.65±0.11	0.66±0.11	0.01±0.11	0.419	0.69±0.01	0.73±0.12	0.04±0.01	0.009*
**Lipids profile**								
High-density lipoprotein (HDL-C)	1.23±0.35	1.20±0.39	-0.03±0.32	0.248	1.17±0.39	1.18±0.39	0.01±0.39	0.388
Low-density lipoprotein (LDL-C)	2.67±0.97	2.71±0.89	0.04±0.95	0.623	2.95±1.03	3.29±0.88	0.34±0.82	<0.001**
Total cholesterol (TC)	4.15±1.06	4.92±1.14	0.77±1.11	<0.001**	3.97±1.05	4.29±1.05	0.32±0.78	<0.001**
Triglycerides (Trig)	1.05±0.47	1.33±0.68	0.28±0.68	<0.001**	1.16±0.70	1.11±0.51	0.05±0.62	0.282
LDL/HDL ratio	2.61±2.51	2.38±1.10	-0.23±2.49	0.210	3.16±3.08	3.07±1.32	-0.09±3.14	0.403
TC/LDL-C ratio	3.69±1.24	4.29±1.50	0.60±1.46	<0.001**	3.71±1.29	3.91±1.29	0.20±1.38	0.119
Trig/LDL-C ratio	0.96±0.51	1.15±0.65	0.19±0.61	<0.001**	1.17±1.05	1.06±0.62	-0.11±1.04	0.183
**Obesity measurements**								
Visceral fat	6.76±2.29	6.41±2.83	-0.35±2.51	0.029*	6.21±1.88	5.05±1.95	-1.16±2.21	<0.001**
Subcutaneous fat	2.28±0.99	2.21±1.12	-0.07±1.07	0.181	0.96±0.51	1.23±1.10	0.27±0.96	0.003*
Waist circumference	94.78±15.49	95.43±14.28	0.64±9.16	0.166	82.62±11.89	83.86±10.46	1.24±8.13	0.108
Hip circumference	109.58±14.77	110.76±15.85	1.18±9.43	0.043*	91.74±9.48	93.87±9.61	2.13±5.68	0.002*
Waist/hip ratio	0.87±0.09	0.86±0.08	-0.01±0.86	0.368	0.90±0.06	0.89±0.07	-0.01±0.08	0.323
Body mass index	31.14±8.05	31.75±7.82	0.61±3.52	0.009*	22.60±4.24	23.06±4.25	-0.43±2.61	0.088
**Blood pressure measurement**								
Systolic blood pressure	126.27±19.69	131.21±22.03	4.94±20.33	<0.001**	126.21±17.71	128.13±20.13	1.93±19.47	0.211
Diastolic blood pressure	81.80±12.90	80.80±11.47	-1.00±12.87	0.035*	79.40±11.02	78.33±10.42	-1.07±10.79	0.209
Pulse	68.28±12.52	71.03±11.64	2.75±14.82	0.006*	64.66±11.73	68.84±11.23	4.17±11.97	0.003*

Data were presented in terms of mean ± standard deviation. P-Value: Significance of the results (*p < 0.05, **p < 0.001). CIMT: carotid intima-media thickness. HDL-C: High-density lipoproteins cholesterol. LDL-C: Low-density lipoproteins cholesterol. TC: total cholesterol. Trig: Triglycerides. T2DM: type 2 diabetes mellitus.

Table S2. Longitudinal estimates of the association of CIMT with CVD risk factors in T2DM group and non-DM controls using linear mixed models.

	T2DM Group			Non-DM Group		
	**Estimates**	**Std error**	**P-Value**	**Estimates**	**Std error**	**P-Value**
**Variables**						
Age	2.20	3.36	<0.001**	0.000	0.002	0.372
**Lipids profile**						
High-density lipoprotein (HDL-C)	0.004	0.001	0.101	0.002	0.004	0.117
Low-density lipoprotein (LDL-C)	4.30	7.18	<0.001**	0.001	0.000	<0.001**
Total cholesterol (TC)	0.000	5.93	0.364	0.000	9.657	0.291
Triglycerides (Trig)	0.003	0.001	0.441	0.001	0.001	0.340
LDL/HDL ratio	0.001	7.13	<0.001**	0.001	0.000	<0.001**
TC/HDL-C ratio	0.00	7.21	0.043*	0.000	8.815	0.024*
Trig/LDL-C ratio	0.004	0.001	0.091	0.000	0.001	0.94
**Obesity measurements**						
Visceral fat	7.102	3.290	0.994	6.475	0.001	0.367
Subcutaneous fat	0.001	0.001	0.963	8.468	0.000	0.479
Waist circumference	9.680	1.45	0.073	2.280	4.292	0.401
Hip circumference	7.394	1.156	0.274	0.000	0.000	0.633
Waist/hip ratio	0.237	0.121	0.001*	0.011	0.002	0.591
Body mass index	9.612	1.669	0.241	9.064	0.000	0.932
**Blood pressure measurement**						
Systolic blood pressure	4.572	6.851	0.019*	1.411	1.232	0.004*
Diastolic blood pressure	7.281	1.914	0.733	6.308	4.315	0.976
Pulse	1.924	2.853	0.877	2.31	0.000	0.920

Estimates represent the effect size for each variable in diabetic and non-diabetic groups. Std Error: Standard Error of the estimate. P-Value: Significance of the results (*p < 0.05, **p < 0.001). HDL-C: High-density lipoprotein cholesterol. LDL-C: Low-density lipoprotein cholesterol. TC: Total cholesterol. Trig: Triglycerides.
